# CRUSE^®^—An innovative mobile application for patient monitoring and management in chronic spontaneous urticaria

**DOI:** 10.1002/clt2.12328

**Published:** 2024-01-03

**Authors:** Sophia Neisinger, Bernardo Sousa Pinto, Aiste Ramanauskaite, Jean Bousquet, Karsten Weller, Martin Metz, Markus Magerl, Emek Kocatürk, Ivan Cherrez‐Ojeda, Ana M. Gimenez‐Arnau, Claudio Alberto S. Parisi, Sabine Altrichter, Luis Felipe Ensina, Laurence Bouillet, Riccardo Asero, Margarida Gonçalo, Carole Guillet, Krzysztof Rutkowski, Jonathan A. Bernstein, Hannah Hardin, Kiran Godse, Zenon Brzoza, Jose Ignacio Larco Sousa, Simon Francis Thomsen, Martijn van Doorn, Michihiro Hide, Young‐Min Ye, Staffan Vanderse, Lāsma Lapiņa, Jonny Peter, Zuotao Zhao, Lianyi Han, Iman Nasr, Heike Rockmann‐Helmbach, Jennifer Astrup Sørensen, Rabia Öztaş Kara, Natalja Kurjāne, Andrii I. Kurchenko, Igor Kaidashev, Vladyslav Tsaryk, Roman Stepanenko, Marcus Maurer

**Affiliations:** ^1^ Urticaria Center of Reference and Excellence (UCARE) Institute of Allergology Charité – Universitätsmedizin Berlin Corporate Member of Freie Universität Berlin and Humboldt‐Universität zu Berlin Berlin Germany; ^2^ Fraunhofer Institute for Translational Medicine and Pharmacology ITMP Allergology and Immunology Berlin Germany; ^3^ Faculty of Medicine MEDCIDS‐Department of Community Medicine Information and Health Decision Sciences University of Porto Porto Portugal; ^4^ CINTESIS‐Center for Health Technology and Services Research University of Porto Porto Portugal; ^5^ RISE‐Health Research Network University of Porto Porto Portugal; ^6^ Department of Dermatology UCARE Koç University School of Medicine Istanbul Turkey; ^7^ Universidad Espíritu Santo Respiralab Research Center Guayaquil Ecuador; ^8^ Department of Dermatology UCARE Hospital del Mar and Research Institute Universitat Pompeu Fabra Barcelona Spain; ^9^ Adult and Pediatric Allergy Sections of the Italian Hospital of Buenos Aires Buenos Aires Argentina; ^10^ Department of Dermatology and Venerology UCARE Kepler University Hospital Linz Austria; ^11^ Faculty of Medicine Center for Medical Research Johannes Kepler University Linz Austria; ^12^ Division of Allergy, Clinical Immunology and Rheumatology Department of Pediatrics Federal University of Sao Paulo Sao Paulo Brazil; ^13^ National Reference Center for Angioedema Internal Medicine Department Grenoble University Hospital Grenoble France; ^14^ Ambulatorio di Allergologia Clinica San Carlo Paderno Dugnano (MI) Italy; ^15^ Faculty of Medicine Coimbra University Hospital University of Coimbra Coimbra Portugal; ^16^ Department of Dermatology University Hospital Zurich Zurich Switzerland; ^17^ Urticaria Clinic St John's Institute of Dermatology Guy's and St Thomas' Hospital London UK; ^18^ Division of Rheumatology, Allergy and Immunology University of Cincinnati College of Medicine Cincinnati Ohio USA; ^19^ Ohio University Heritage College of Osteopathic Medicine Cleveland Ohio USA; ^20^ Department of Dermatology Dr D Y Patil Medical College and Hospital Navi Mumbai India; ^21^ Division of Allergology Department of Internal Diseases Institute of Medical Sciences University of Opole Opole Poland; ^22^ Department of Allergy UCARE Clinica San Felipe Lima Peru; ^23^ Department of Biomedical Sciences Department of Dermatology Bispebjerg Hospital University of Copenhagen Copenhagen Denmark; ^24^ Department of Dermatology UCARE Erasmus MC Rotterdam The Netherlands; ^25^ Department of Dermatology Hiroshima City Hiroshima Citizens Hospital Hiroshima Japan; ^26^ Department of Allergy and Clinical Immunology Ajou University School of Medicine Suwon Korea; ^27^ Dermatologie/Venerologie Bundeswehrkrankenhaus Berlin Akademisches Lehrkrankenhaus der Charité Berlin Germany; ^28^ Allergic Diseases Diagnosis and Treatment Center Riga Stradins University Pauls Stradins Clinical University Hospital Riga Latvia; ^29^ ACARE Centre Division of Allergy and Clinical Immunology Department of Medicine Groote Schuur Hospital University of Cape Town Cape Town South Africa; ^30^ Department of Dermatology and Venerology Beijing Key Laboratory of Molecular Diagnosis on Dermatoses National Clinical Research Center for Skin and Immune Diseases Peking University First Hospital Beijing China; ^31^ Greater Bay Area Institute of Precision Medicine (Guangzhou) School of Life Sciences Fudan University Shanghai China; ^32^ Department of Clinical Immunology and Allergy Royal Hospital Muscat Oman; ^33^ Department of Dermatology/Allergology University Medical Centre Utrecht Utrecht University Utrecht The Netherlands; ^34^ Department of Dermatology Sakarya University Faculty of Medicine Sakarya Turkey; ^35^ Department of Clinical and Laboratory Immunology, Allergology and Medical Genetics Bogomolets National Medical University Kyiv Ukraine; ^36^ Poltava State Medical University Poltava Ukraine; ^37^ Department of Dermatology and Venereology with Cosmetology Course Bogomolets National Medical University Kyiv Ukraine

**Keywords:** chronic urticaria self evaluation (CRUSE^®^) app, digital tools, mHealth apps, patient‐reported outcome measures, wheals and angioedema

## Abstract

**Background:**

Chronic spontaneous urticaria (CSU) is unpredictable and can severely impair patients' quality of life. Patients with CSU need a convenient, user‐friendly platform to complete patient‐reported outcome measures (PROMs) on their mobile devices. CRUSE^®^, the Chronic Urticaria Self Evaluation app, aims to address this unmet need.

**Methods:**

CRUSE^®^ was developed by an international steering committee of urticaria specialists. Priorities for the app based on recent findings in CSU were defined to allow patients to track and record their symptoms and medication use over time and send photographs. The CRUSE^®^ app collects patient data such as age, sex, disease onset, triggers, medication, and CSU characteristics that can be sent securely to physicians, providing real‐time insights. Additionally, CRUSE^®^ contains PROMs to assess disease activity and control, which are individualised to patient profiles and clinical manifestations.

**Results:**

CRUSE^®^ was launched in Germany in March 2022 and is now available for free in 17 countries. It is adapted to the local language and displays a country‐specific list of available urticaria medications. English and Ukrainian versions are available worldwide. From July 2022 to June 2023, 25,710 observations were documented by 2540 users; 72.7% were females, with a mean age of 39.6 years. At baseline, 93.7% and 51.3% of users had wheals and angioedema, respectively. Second‐generation antihistamines were used in 74.0% of days.

**Conclusions:**

The initial data from CRUSE^®^ show the wide use and utility of effectively tracking patients' disease activity and control, paving the way for personalised CSU management.

## 1 INTRODUCTION

Chronic spontaneous urticaria (CSU) is a common, debilitating immunological condition that manifests in the skin. It is characterised by itchy recurrent wheals and/or angioedema that recur for longer than 6 weeks.^1^, ^2^ The signs and symptoms of CSU develop spontaneously, although drugs, infection^3^ and high stress levels^4^ can exacerbate the disease activity.^5^ Many patients experience a protracted disease course that can last several years.^1^, ^6^ Due to its unpredictable nature, incapacitating symptoms, and associated comorbidities, CSU severely impairs patients' quality of life (QoL) and poses relevant economic impacts.^7^, ^8^, ^9^, ^10^ Because of the random, fluctuating occurrence of wheals and angioedema, patients can fear disease exacerbation, avoiding social events and activities they once enjoyed and missing work and school days. CSU substantially affects objective functioning and subjective well‐being,^8^, ^9^ as many patients have daily or almost‐daily signs and symptoms,^11^ resulting in the feeling of losing control over their lives.^12^, ^13^ Patients can also experience sleep impairment,^14^ anxiety, and depression,^15^ all of which have been shown to increase with worsening disease activity.^9^ Patients with CSU often visit several physicians or specialists searching for the cause and appropriate treatment for their condition.

Using standardised, validated patient‐reported outcome measures (PROMs) is fundamental for managing and monitoring responses to treatment in patients with CSU and is recommended in the current international guidelines.^1^, ^16^ PROMs are powerful, self‐reported measurements collected directly from patients to express their perception of disease, focusing on patient‐centred care and what matters most to patients, a perspective often missed by physicians.^17^ PROMs compare different treatment modalities, and data can be leveraged using mobile tools and sophisticated data analysis. PROMs used in CSU measure the daily or weekly occurrence of wheals and angioedema, levels of disease control, and CSU impact on QoL; Table 1 shows the most common PROMs used to evaluate CSU.

**TABLE 1:** Commonly used PROMs in CSU assessment.

PROM name	What it assesses	Details	PROM scale and number of questions	Recall period
Urticaria activity score (UAS)^18^, ^19^	The daily activity of wheals and pruritus	Because of the frequently fluctuating nature of CSU, higher reliability is achieved when the overall disease activity is measured once daily for several days	Score is 0–3 for wheals/hives and 0–3 for itch over 24 h The maximum total score of the UAS7, the weekly sum score is 42 (range 0–42) Five score bands: 0, 1–6, 7–15, 16–27, 28–42, reflecting urticaria‐free to severe disease activity	24 h
Angioedema activity score (AAS)^20^	The activity of skin swellings	It consists of one screening question (angioedema in the last 24 h: Yes/no) and five follow up questions with four answer options for each item (minimum score per day is 0 and maximum score is 15)	One + five questions with four answer options (scored 0–3) Total daily score is 0–15 The AAS28 reflects angioedema activity over 4 weeks Score bands are available	24 h
Urticaria control test (UCT)^21^, ^22^	The level of disease control and response to current treatment	A score of 16 indicates complete disease control. A score of <12 indicates poorly controlled disease, and a score ≥12 identifies patients with well‐controlled CSU	Four‐item tool with clearly defined parameters for patients with well‐controlled versus poorly controlled disease	Four weeks (UCT) or 7 days (UCT7)
Angioedema control test (AECT)^23^, ^24^	The level of disease control in patients with recurrent angioedema	A score of <10 indicates poorly controlled angioedema, and a score ≥10 identifies patients with well‐controlled angioedema	Four‐item tool with clearly defined parameters for patients with well‐controlled versus poorly controlled disease	Four weeks (AECT‐4wk) or 3 months (AECT‐3mo)

Abbreviations: CSU, chronic spontaneous urticaria; PROMs, patient‐reported outcome measures.

Patients with CSU face several challenges in managing their condition, including the need for multiple physician visits, time‐consuming treatment schedules, and access to appropriate expert care.^25^, ^26^ PROMs can help with these challenges, but they appear to be severely underused in chronic urticaria, with many physicians not utilising them.^27^ The main barriers to wider PROM use include time constrains, a lack of integration into clinical systems and the unavailability for certain age groups.^27^ Irrespective of age, patients often prefer mobile health (mHealth) apps to paper documentation when using PROMs. There is therefore an increasing demand for healthcare solutions capable of overcoming these barriers and streamlining the use of PROMs in the treatment process at various levels of healthcare to enable coordinated, proactive patient care.^28^ Patients' use of real‐time, user‐friendly digital tools before their visits (such as mHealth apps) could counteract time restraints and transform patients' perceptions of PROMs, streamlining disease management.

Following the identified unmet needs for the treatment of patients with CSU,^29^ the global network of Urticaria Centers of Reference and Excellence (UCARE)^30^ developed the CRUSE^®^ (Chronic Urticaria Self Evaluation) app to create a user‐friendly platform allowing patients to complete PROMs on their mobile devices in a convenient way and to make these results easily available to their treating physician. The advancement of digital health and mHealth services reflects a demand and change in attitude from patients to allow better engagement and self‐management outside of the doctor's office.^31^ One year after the launch of CRUSE^®^, we herein describe the development and features of the app and its initial results.

## 2 METHODS

### 2.1 The inception of CRUSE^®^: The UCARE CURICT project and its key findings on apps for CSU

In July 2021, the Chronic Urticaria Information and Communication Technologies (CURICT [ICT]) project explored the use of ICT in patients diagnosed with CSU.^32^, ^33^ The CURICT project, conducted by the UCARE network,^29^ showed that most patients with CSU were very or extremely interested in using an app to monitor disease activity and control.^32^ Subsequently, an algorithm was utilised to search app stores across 16 countries to identify available apps that help patients track their CSU signs and symptoms.^29^ This project found that iOS and/or Android apps for the self‐evaluation of CSU are extremely limited in number (only five apps were available), function, and geographical reach.^29^ UCARE analysed the apps available to self‐assess urticaria and subsequently created a new, user‐friendly, global tool.

### 2.2 Development of the CRUSE^®^ app

CRUSE^®^ was developed by an international steering committee of urticaria specialists from UCAREs with the partnership of Professor Jean Bousquet, who was responsible for pioneering the MASK‐air app.^34^ The steering committee, together with a CRUSE^®^ core team, was tasked with defining objectives and key requirements for the app based on recent findings in the field of CSU, including those from the UCARE CURICT project, so that the app would adequately address the critical needs of the urticaria community.

Patient empowerment was a major goal of CRUSE^®^. The app enables patients to track their symptoms and medication use, take and send photographs, and record their condition over time. This information is transmitted securely via email to their physician, providing valuable, real‐time insights. Additionally, the app will offer patients many educational resources and support tools to aid in better understanding and management of their condition, a feature which is currently in development. For instance, patients will be able to obtain information on the causes of CSU and tips for managing their symptoms.

### 2.3 Features of CRUSE^®^

Users can download the CRUSE^®^ app to their phones, with links provided on the CRUSE^®^ website (https://cruse‐control.com), in Google Play or the Apple App Store. After registration, patients can complete their CRUSE^®^ app profile, providing information on their age, sex, disease onset, triggers, medication, and CSU characteristics (presence of wheals and/or angioedema).

Patients can also enter a study identification (ID) number when registering, thus enabling investigators to use the data captured by CRUSE^®^ in specific scientific projects (if the CRUSE^®^ steering committee has accepted this and if approved by an Institutional Review Board), potentially connecting CRUSE^®^ data with other data sources. This function allows patients to enter their CURE registry ID, connecting the app to the worldwide urticaria registry.^32^

The app has a quick response code which enables patients to share data with their physician and a function to share via email. This feature allows patients to easily share their CSU control level between consultations and facilitates communication when patients and physicians do not share the same language. For instance, a key feature of CRUSE^®^ is to help facilitate communication between Ukrainian refugees and their physicians, as seen in the Ukrainian Citizen and Refugee Electronic Support in Respiratory, Allergy, Immunology and Dermatology (UCRAID) project^35^ (www.ucraid.com; see discussion for more details).

### 2.4 PROMs used in the CRUSE^®^ app

**FIGURE 1:**
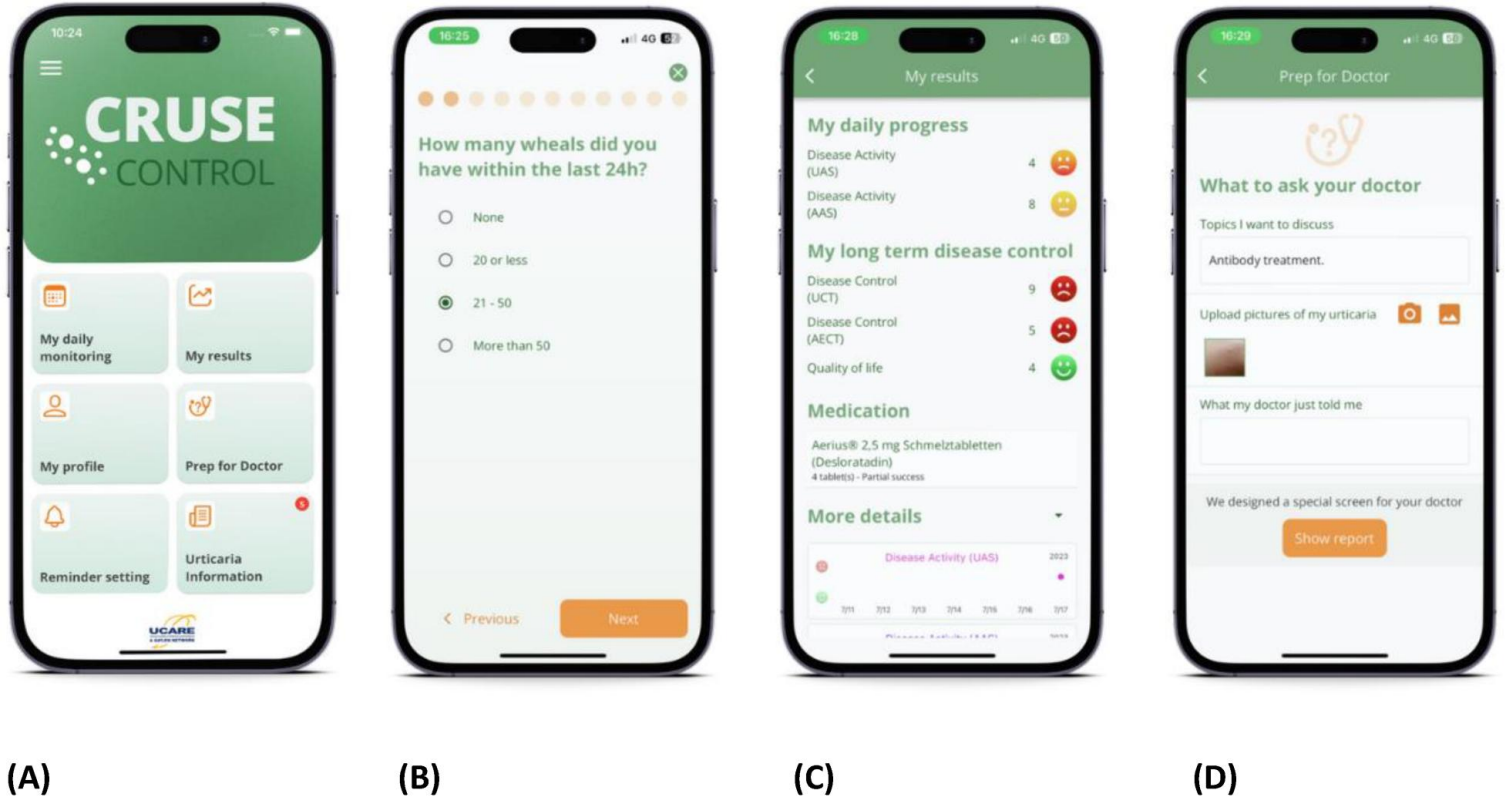
Screenshots of the CRUSE^®^ app. Screenshots of the CRUSE^®^ app. (A) CRUSE^®^ home screen, (B) an example of the daily questions, (C) the app shows patients their personal results, and (D) the report is prepared to be sent to the physician.

CRUSE^®^ comprises validated urticaria PROMs to assess disease activity and control, namely the urticaria activity score (UAS),^18^, ^19^ angioedema activity score (AAS),^20^ urticaria control test (UCT),^21^, ^22^ and angioedema control test (AECT^23^, ^24^, ^36^, ^37^; see Table 1 for details). It also includes the EQ‐5D visual analogue scale (EQ‐5D VAS).^38^ This quantitative measure reflects the patients' judgement on how their health is on that day, ranging from ‘the best health you can imagine’ to ‘the worst health you can imagine’. PROM data are collected daily for UAS, AAS, urticaria/angioedema impact on work or school performance (assessed using VAS); they are collected monthly for UCT and AECT (Figure 1).

PROMs are displayed in the app according to the information entered by the patient in their profile; for example, if a patient has no angioedema, the AAS and AECT will not be displayed. The app automatically calculates the PROM scores, showing them in an easily understandable way, and the patient can send a secure report, including current medication and pictures, to their treating physician. These PROMs are available in the countries where CRUSE^®^ was launched in the respective languages. A key consideration was that the app remained user‐friendly and engaging. To achieve this, only the most important PROMs and questions were selected to avoid overwhelming the user.

### 2.5 Medication list used in the CRUSE^®^ app

In clinical practice, it is crucial to have patients actively respond to PROMs and be aware of the medications they are taking. The CRUSE^®^ app can help physicians encourage their patients to actively participate in the management of CSU. A CRUSE^®^ champion, that is, an experienced urticariologist and UCARE physician, from each country created an urticaria medication list for that respective country, tailored to the medications available. The list might also include off‐label medications.

### 2.6 Ethics and data storage

All data from the CRUSE^®^ study are anonymised. Upon registration, the user agrees to the app's terms and conditions of use and the data fair use agreement, which allows the anonymous use of its data for research purposes. Users must be 18 years old or over to use the app, as stated in the terms and conditions. Further individual patient consent is unnecessary for analysing anonymised data in CRUSE^®^. The data are stored in CloudVPS in the Netherlands according to ISO 27001 and ISO 13485 standards and meets Regulation (EU) 2016/679 on General Data Protection. Institutional Review Board approval was not required.

### 2.7 Data analysis

In the current analysis, we assessed all data provided by all users (except test users) from the 1 July 2022 to the 30 June 2023. We described CRUSE^®^ app users according to their baseline age, sex, country, CSU type, and triggers. Categorical variables are described using absolute and relative frequencies, while continuous variables are described using means, standard deviations, medians, and interquartile ranges. All analyses were conducted using Software R—version 4.3.0. CRUSE is an open‐ended, ongoing project with no planned end date.

### 2.8 The CRUSE^®^ app, Website and tester Function for physicians

After the first few months of launching the CRUSE^®^ app, feedback from colleagues was essential to adapt the app to meet the needs of patients and physicians; thus, a tester function was initiated to differentiate testers from patients. To try out CRUSE^®^, testers can visit the website (https://cruse‐control.com), download the app, and use the tester function. To use this tester function, users register as a patient and click ‘About this app’ on the upper lefthand side of the main menu. The lower righthand corner states the app version (e.g., V.1.0.21#), and clicking the version number six times activates the test mode, with the word ‘Tester’ shown next to the number (e.g., V.1.0.21#—tester).

## 3 RESULTS

### 3.1 CRUSE^®^ dissemination

CRUSE^®^ was initially launched in Germany in March 2022 as a freely available app for patients with CSU. It was then progressively launched in 16 additional countries, to bring the app to all patients with CSU. National CRUSE^®^ champions, that is, experienced urticariologists and UCARE physicians, helped launch the app in their respective countries by providing translations, a local CSU medication list, raising awareness, and increasing knowledge of the app with physicians and patients. CRUSE^®^ champions played, and continue to play, a key role in actively promoting the app in their respective countries, including educating other physicians on how to effectively utilise it in their clinics, talking to patient groups, and using the app themselves.

CRUSE^®^ has been specifically launched in 17 countries, including Germany, Austria, Switzerland, the UK, Spain, Italy, Portugal, France, Turkey, Poland, Ecuador, Peru, Brazil, Argentina, Latvia, Ukraine, and Denmark. In all these countries, CRUSE^®^ has been adapted to the respective local languages and displays a country‐specific list of available medications. English and Ukrainian versions are also available worldwide to give patients with CSU faster access to CRUSE^®^ in countries where it has not yet been launched. The English version provides content entirely in English, irrespective of the patient's location. It includes a list of medications commonly used for urticaria worldwide.^1^ The Ukrainian version is part of the UCRAID project;^35^ (see discussion for more details).

### 3.2 Characteristics of CRUSE^®^ users

**FIGURE 2:**
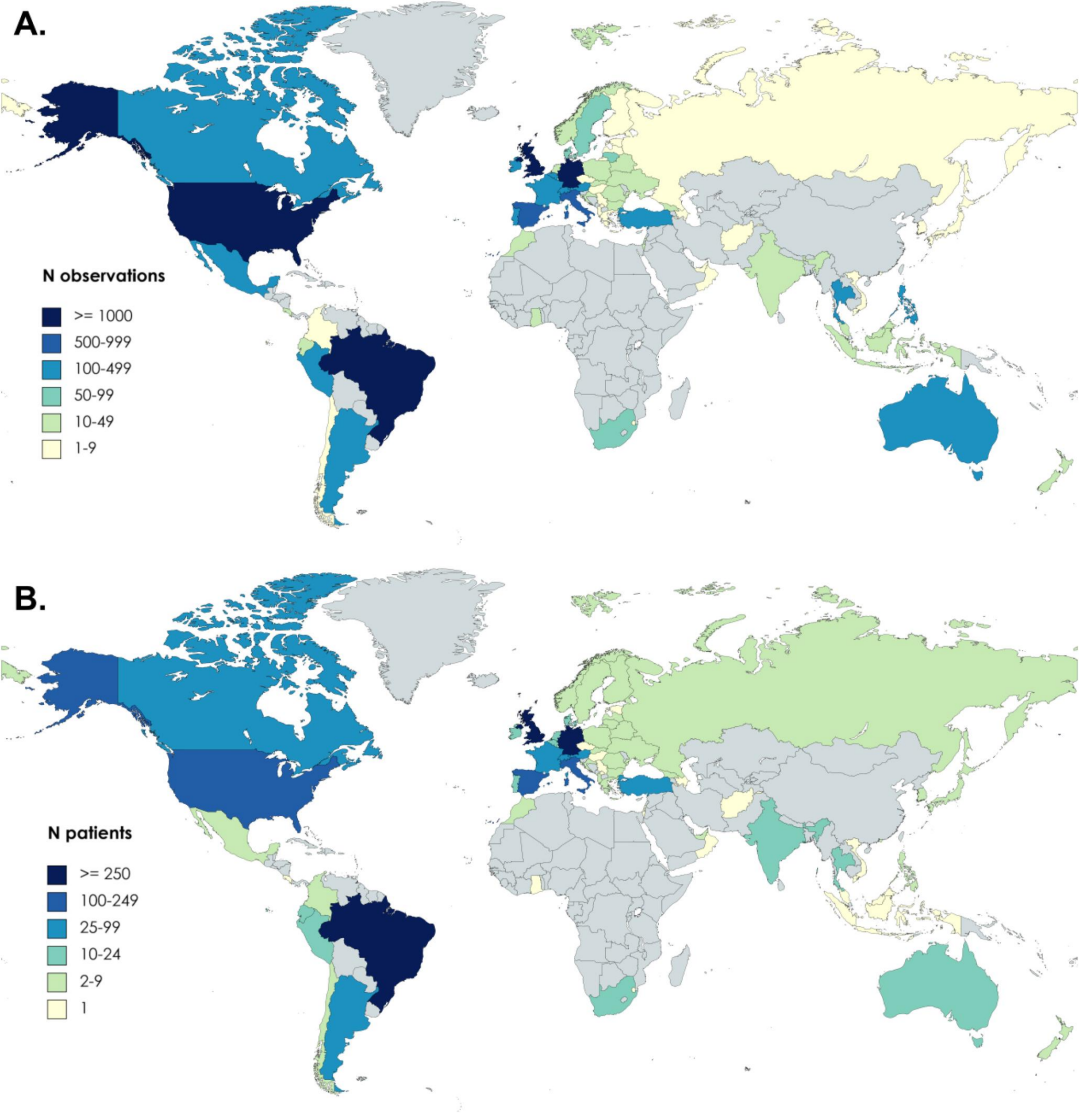
Geographical distribution of CRUSE^®^ app observations (A) and users (B). The worldwide reach of the CRUSE^®^ app is shown by (A) individual observations and (B) total CRUSE^®^ users.

**FIGURE 3:**
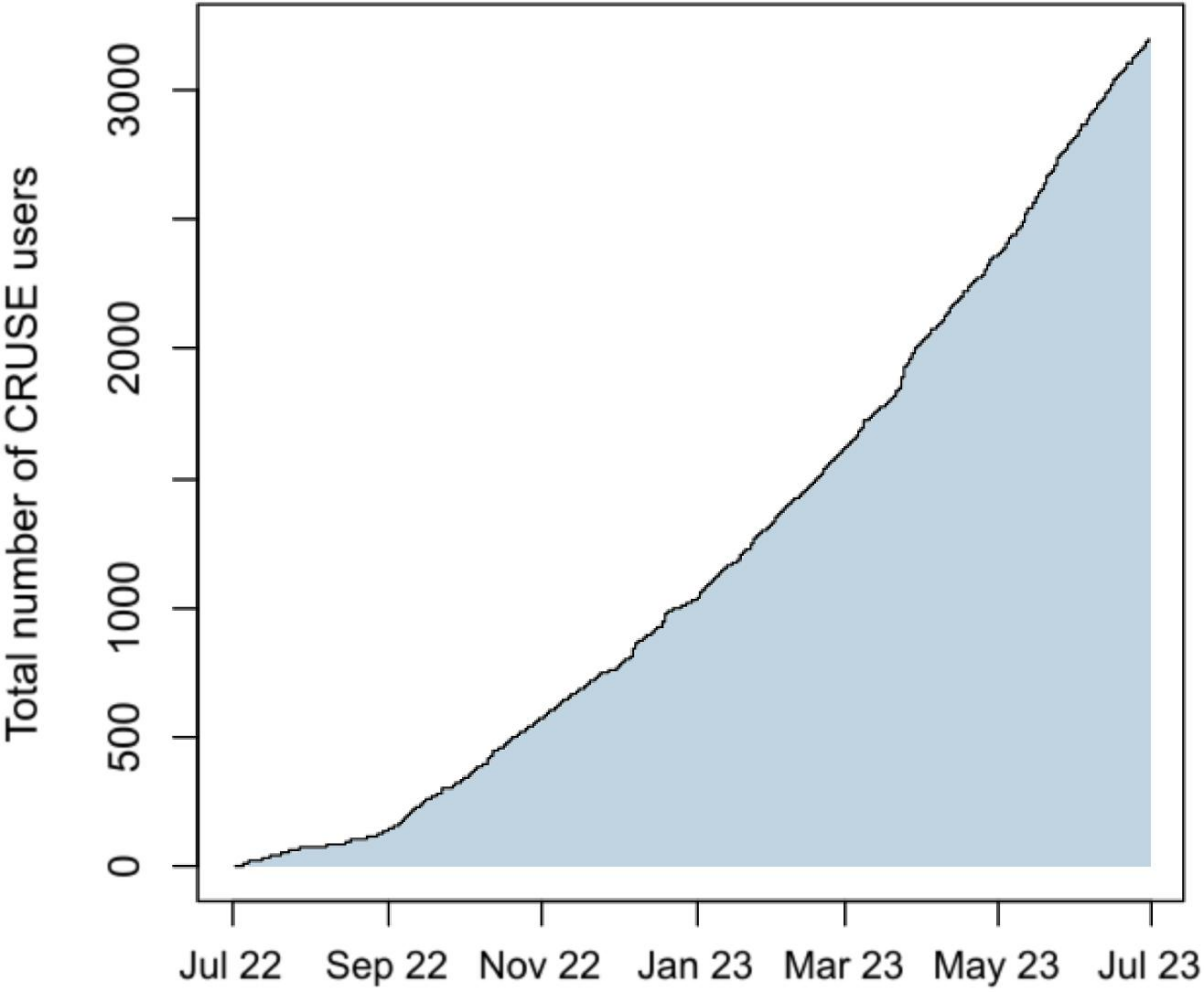
Total downloads of the CRUSE^®^ app from July 2022 to July 2023. The total number of downloads of the CRUSE^®^ app from July 2022 to July 2023.

As of the 18 September 2023, CRUSE^®^ had over 5000 users. This paper presents data from 2540 users, including 25,710 individual days of documentation from 1 year of CRUSE^®^: 1 July 2022 to the 30 June 2023 (Figures 2 and 3). Overall, 72.7% of users were female and the mean user age was 39.6 (SD: 14.6) years (Table 2). Most users were from Germany (39.6%) or other European countries (31.4%; Table 2). Almost all users (93.7%) reported wheals, and 51.3% reported angioedema. Triggers for CSU exacerbation were reported by 61.5% of users, the most common ones being stress (30.7%), rubbing the skin (24.4%) and high temperatures (20.9%).

**TABLE 2:** Characteristics of CSU patients who use CRUSE^®^.

Patient characteristic	Patients, *N* (%)
Sex	
Females	1846 (72.7)
Males	666 (26.2)
Other	28 (1.1)
Age in years—Mean (SD)	39.6 (14.6)
Country	
Germany	1005 (39.6)
Other European countries	798 (31.4)
Other countries	737 (29.0)
CSU manifests with wheals—*N* (%)	2379 (93.7)
CSU manifests with angioedema—*N* (%)	1302 (51.3)
Existence of triggers	1562 (61.5)
Stress	780 (30.7)
Rubbing of skin	621 (24.4)
High temperature	531 (20.9)
Food	453 (17.8)
Exercise	312 (12.3)
Medication	286 (11.3)
Cold temperature	265 (10.4)
Infection	256 (10.1)
Medication use	
Anti‐H_1_ of second generation	1928 (75.9%)
Monoclonal antibody	500 (19.7%)
Omalizumab	488 (19.2%)
Mepolizumab	3 (0.1%)
Dupilumab	3 (0.1%)
Benralizumab	3 (0.1%)
Secukinumab	3 (0.1%)
Corticosteroid	221 (8.7%)
Anti‐H_1_ of first generation	147 (5.8%)
Leukotriene receptor antagonist	138 (5.4%)
Anti‐H_2_	112 (4.4%)
Calcineurin inhibitor	17 (0.7)
Antimalarial	6 (0.2)
Sulfone	3 (0.1)

*Note*: Data are derived from the information provided by 2540 patients during registration of their patient profiles.

Abbreviations: CSU, chronic spontaneous urticaria; SD, Standard‐deviation.

### 3.3 Medication use patterns

Most CRUSE^®^ app users reported at least 1 day of second‐generation antihistamine use (75.9%). Monoclonal therapeutic antibodies (19.7%) and corticosteroids (8.7%) were the next most frequently used therapies reported for at least 1 day (Table 2). Considering all days during the first year after the CRUSE app^®^ launch, the use of second‐generation antihistamines was reported in 19,025 out of 25,710 days (74.0%), with only 848 out of 25,710 days (3.3%) using first‐generation antihistamines (Table 3). Corticosteroid use was reported in 1244 out of 25,710 days (4.8%). Leukotriene receptor antagonists and anti‐H_2_ medication were used in 2058 days (8.0%) and 1317 days (5.1%) out of 25,710 days, respectively, and monoclonal antibodies were used in 1275 out of 25,710 days (5.0%; most [98.4%] of them were omalizumab, with the remaining days including benralizumab, dupilumab, mepolizumab and secukinumab).

**TABLE 3:** Occurrence of wheals, angioedema, and both, as well as use of medication documented by the use of CRUSE^®^ during the first year after launch.

Event	Days of occurrence, *N* (%)
Wheals	14,288 of 25,710 (55.6)
Angioedema	4173 of 25,710 (16.2)
Wheals, no angioedema	10,851 of 25,710 (42.2)
Angioedema, no wheals	699 of 25,710 (2.7)
Wheals and angioedema	3437 of 25,710 (13.4)
Use of anti‐H_1_ of second generation	19,025 of 25,710 (74.0)
Use of leukotriene receptor antagonist	2058 of 25,710 (8.0)
Use of anti‐H_2_	1317 of 25,710 (5.1)
Use of monoclonal antibody^a^	1275 of 25,710 (5.0)
Use of corticosteroid	1244 of 25,710 (4.8)
Use of anti‐H_1_ of first generation	848 of 25,710 (3.3)
Use of antimalarial	144 of 25,710 (0.6)
Use of calcineurin inhibitor	106 of 25,710 (0.4)
Use of sulfone	10 of 25,710 (0.04)

^a^ Includes 1255 days of omalizumab use, 7 days of dupilumab use, 5 days of mepolizumab use, 4 days of benralizumab use and 4 days of secukinumab use.

## 4 DISCUSSION

The launch of CRUSE^®^, the first mHealth app to comprehensively monitor CSU signs and symptoms, impact, and control, marks a milestone in managing CSU. This exploratory study demonstrated that within one year, 2540 users could utilise the CRUSE^®^ app to record their baseline characteristics and track the progression of their disease. These encouraging results demonstrate the value of this simple, user‐friendly smartphone platform, which has been tested by patients worldwide, indicating the demand for patients to empower themselves with better knowledge and control their disease. CRUSE^®^ aims to encourage active patient engagement and foster stronger communication between patients and physicians. The importance of informed patients taking an active role in managing chronic conditions must be considered; knowledgeable patients are better equipped to make appropriate treatment decisions and adhere to strategies set out by their physicians.^39^

Baseline data from CRUSE^®^ were similar to those reported in the literature; most CRUSE^®^ users were female (72.7%), and the average age was 39.6 years. More than half of the CRUSE^®^ users, 51.3%, reported angioedema. These results closely align with data already published from CURE and other real‐world studies, where 72.4% of patients were female, the average age was 43.0 years,^40^ and the rate of angioedema was 58.5% in patients with CSU.^9^

CRUSE^®^ can potentially play a role in shaping future CSU treatment paradigms, namely by allowing UCT and AECT scores to be directly sent to physicians. As such, physicians can determine whether any treatment changes are required in real‐time without the need for lengthy appointment waiting times. This aligns with the current guidelines in which it is recommended that a patient's treatment is adjusted depending on their UCT score; patients with a UCT <12 should be treated with 1–4 times second‐generation antihistamines for >7–28 days or omalizumab for >3 months, those with a UCT = 12–15 should continue and aim to optimise their current therapy, and those with a UCT = 16 may consider stepping down their treatment.^1^ Of note, other mHealth technologies, such as MASK‐air^®^, have already demonstrated great success in monitoring patients with allergic rhinitis and asthma.^41^

Further analyses will determine the app's role in (i) improving CSU care, (ii) advancing further knowledge on the disease, and (iii) allowing for faster diagnoses. Regarding the latter, one of the current challenges in CSU is the considerable delay between diagnosis and specialist referral, partly due to inadequate knowledge about CSU and insufficient use of PROMs among primary and secondary care physicians.^27^ Diagnostic delays are often associated with costly and unnecessary investigations and treatments.

### 4.1 The potential of CRUSE^®^—The UCRAID project

In addition to the functions already discussed, the CRUSE^®^ app forms part of the UCRAID action plan. UCRAID aims to provide Ukrainian refugees diagnosed with CSU access to the CRUSE^®^ app. Patients can download the app in Ukrainians around the world regardless of their location, offering crucial support during a vulnerable period in their lives and demonstrating the commitment of UCARE to global accessibility.

An estimated eight million Ukrainians have fled the war and taken refuge in the European Union, with at least 15% (over one million) experiencing asthma, allergic rhinitis, and/or urticaria.^35^ The magnitude of the problem is considerable, and refugees pose several challenges to the recipient countries. The population includes a heterogeneous and vulnerable group with complex health needs for whom physicians often provide care.^42^ Language assistance in non‐native speaking individuals is especially important in healthcare due to the sensitive issues involved and the technical language required when communicating medical terminology.^43^

We expect the UCRAID project to benefit Ukrainian refugees worldwide by engaging them and helping us understand their barriers to providing person‐centred care for chronic diseases. For Ukrainian refugees with CSU, CRUSE^®^ provides a user‐friendly mHealth app in their native language to improve symptom control and communication with physicians abroad, ultimately lowering disability. Engagement with physicians digitally and in person also has the potential to reduce emergency visits and hospitalisations, thus saving costs. Additionally, the deployment of UCRAID will serve as a prototype for other chronic diseases, such as chronic obstructive pulmonary disease or cardiovascular diseases.

### 4.2 Potential challenges of CRUSE^®^

The UCARE CURICT study identified that one potential challenge could lie in the uptake of the CRUSE^®^ app amongst patients unfamiliar with newer technologies.^32^ This is partly because these patients may have less confidence or knowledge of using mHealth apps and are unwilling to adopt technologies, preferring face‐to‐face consultations with their physicians.^44^ Additionally, in some rural or poorer areas, mobile device access could be more limited due to 5G or Wi‐Fi issues, so patients may not have the tools to access CRUSE^®^.

### 4.3 Strengths and limitations

There are potential inherent biases when collecting data via any app, as we must rely on the information self‐reported by the patient. For instance, we do not know whether all users have physician‐diagnosed CSU (or misdiagnosed urticaria), and certain age groups may use the app more frequently, creating a bias in the age distribution. Additionally, the occurrence of another skin condition causing itch in a patient could result in inaccurately elevated scores in the PROMs, as it would not solely reflect the symptoms of urticaria. Moreover, patients might stop using the app over time, especially if they feel better, lose interest, or use it less because of other limiting medical conditions. This could lead to a biased sample because those who continue using the app may have a different disease impact than those who stop using it. In addition, those days patients use the CRUSE^®^ app may be systematically different from the remaining days that is, patients may use CRUSE^®^ more often when feeling worse.

### 4.4 Plans and development of the CRUSE^®^ app

As part of the ongoing development, there are plans to include a larger repository of easily accessible patient education features within CRUSE^®^. Future iterations may benefit from integrating artificial intelligence and machine learning algorithms to analyse patient data and personalise recommendations based on individual disease patterns and treatment responses. CRUSE^®^ also enables large numbers of patients to be reached, and future versions will be used to inform users about upcoming new treatment options and novel insights on the causes, triggers, comorbidities, and consequences of CSU.

Because CRUSE^®^ seamlessly connects to the global CURE database, a robust directory for researchers and healthcare providers is continually being created to analyse data from the patient's unique perspective, helping us better understand CSU and facilitate data sharing.^45^ CRUSE^®^ will be used for post‐marketing studies and real‐world research. The aim is that this will convey insights into CSU progression and answer some of the currently unknown questions about CSU. Some of the questions we predict CRUSE^®^ will help establish include quantifying the impact of CSU on QoL, identifying factors influencing QoL, assessing the impact of CSU on work and school productivity and the associated costs, assessing medication adherence, and assessing treatment efficacy and safety in routine clinical practice. CRUSE^®^ also aims to provide photo documentation, evaluate the socioeconomic impacts, and provide a data repository for research projects, making assessing disease activity and control easier and more convenient.

## 5 CONCLUSIONS

Digital healthcare provides a unique opportunity to deliver effective and sustainable management of chronic conditions in different settings outside the physician's office. The initial data from CRUSE^®^ show that the urticaria community has widely accepted the app, and it can be used effectively to track patients' disease activity and control. CRUSE^®^ may help pave the way for a personalised, patient‐centred approach to CSU management.

## AUTHOR CONTRIBUTIONS

**Sophia Neisinger**: Conceptualization (equal); formal analysis (equal); funding acquisition (equal); investigation (equal); methodology (equal); project administration (equal); resources (equal); writing – review & editing (equal). **Bernardo Sousa Pinto**: Data curation (equal); formal analysis (equal); investigation (equal); methodology (equal); writing – review & editing (equal). **Aiste Ramanauskaite**: Conceptualization (equal); investigation (equal); methodology (equal); project administration (equal); writing – review & editing (equal). **Jean Bousquet**: Investigation (equal); project administration (equal); writing – review & editing (equal). **Karsten Weller**: Investigation (equal); project administration (equal); writing – review & editing (equal). **Martin Metz**: Investigation (equal); project administration (equal); writing – review & editing (equal). **Markus Magerl**: Investigation (equal); project administration (equal); writing – review & editing (equal). **Emek Kocatürk**: Investigation (equal); project administration (equal); writing – review & editing (equal). **Ivan Cherrez‐Ojeda**: Investigation (equal); project administration (equal); writing – review & editing (equal). **Ana M. Gimenez‐Arnau**: Resources (equal); writing – review & editing (equal). **Claudio Alberto S. Parisi**: Resources (equal); writing – review & editing (equal). **Sabine Altrichter**: Resources (equal); writing – review & editing (equal). **Luis Felipe Ensina**: Resources (equal); writing – review & editing (equal). **Laurence Bouillet**: Resources (equal); writing – review & editing (equal). **Ricardo Asero**: Resources (equal); writing – review & editing (equal). **Margarida Goncalo**: Resources (equal); writing – review & editing (equal). **Carole Guillet**: Resources (equal); writing – review & editing (equal). **Krzysztof Rutkowski**: Resources (equal); writing – review & editing (equal). **Jonathan Bernstein**: Resources (equal); writing – review & editing (equal). **Hannah Hardin**: Writing – review & editing (equal). **Kiran Godse**: Resources (equal); writing – review & editing (equal). **Zenon Brzoza**: Resources (equal); writing – review & editing (equal). **Jose Ignacio Larco Sousa**: Resources (equal); writing – review & editing (equal). **Simon Francis Thomsen**: Resources (equal); writing – review & editing (equal). **Martijn van Doorn**: Resources (equal); writing – review & editing (equal). **Michihiro Hide**: Resources (equal); writing – review & editing (equal). **Young‐Min Ye**: Resources (equal); writing – review & editing (equal). **Staffan Vandersee**: Writing – review & editing (equal). **Lāsma Lapiņa:** Resources (equal); writing – review & editing (equal). **Jonny Peter**: Resources (equal); writing – review & editing (equal). **Zuotao Zhao**: Writing – review & editing (equal). **Lianyi Han**: Writing – review & editing (equal). **Iman Nasr**: Resources (equal); writing – review & editing (equal). **Heike Rockmann‐Helmbach**: Resources (equal); writing – review & editing (equal). **Jennifer Astrup Sørensen**: Resources (equal); Writing – review & editing (equal). **Rabia Öztaş Kara**: Resources (equal); writing – review & editing (equal). **Natalja Kurjane**: Resources (equal); writing – review & editing (equal). **Andrii I. Kurchenko**: Writing – review & editing (equal). **Igor Kaidashev**: Writing – review & editing (equal). **Vladyslav Tsaryk**: Writing – review & editing (equal). **Roman Stepanenko**: Writing – review & editing (equal). **Marcus Maurer**: Conceptualization (equal); formal analysis (equal); funding acquisition (equal); investigation (equal); methodology (equal); project administration (equal); resources (equal); supervision (equal); writing – review & editing (equal).

## CONFLICT OF INTEREST STATEMENT

**J. Bousquet** has received an honorarium for lectures or advisory boards from AstraZeneca, GlaxoSmithKline, Meda, Menarini, Novartis, Uriach, and Viatris. He is a partial shareholder of Kyomed Innov and MASK‐air SAS. **K. Weller** or recently was a speaker and/or advisor for and/or has received research funds from Moxie, Novartis, Pharvaris, Shire, Takeda, and Uriach. **M. Metz** is or recently was a speaker and/or consultant for Amgen, AstraZeneca, Argenx, Celldex, Celltrion, Escient, Jasper Therapeutics, Novartis, Pharvaris, Regeneron, Sanofi, and ThirdHarmonicBio. **M. Magerl** is an advisor for Moxie. **E. Kocatürk** has been a speaker and has served on advisory boards for Novartis, Menarini, LaRoche Posey, Sanofi, Bayer, Abdi İbrahim, and Pfizer. **I. Cherrez‐Ojeda** is or recently was a speaker for Sanofi Aventis and Megalabs. **A. M. Gimenez‐Arnau** or recently was a speaker and/or advisor for and/or has received research funding from Almirall, Amgen, AstraZeneca, Avene, Celldex, Escient Pharmaceuticals, Genentech, GlaxoSmithKline, Instituto Carlos III‐ FEDER, Leo Pharma, Menarini, Mitsubishi Tanabe Pharma, Novartis, Sanofi–Regeneron, Servier, Thermo Fisher Scientific, and Uriach Pharma/Neucor. **S. Altrichter** has no conflicts of interest in relation to this manuscript. Outside it, she has conducted studies for/was an advisor for/was a speaker for AstraZeneca, Allakos, ALK, Biocryst, CSLBehring, LeoPharma, Moxie, Novartis, Sanofi, Takeda, and Thermofisher. **L. F. Ensina** has no conflicts of interest in relation to this manuscript. He has conducted studies for Novartis, Amgen, and Sanofi, has been a speaker for Novartis, Sanofi and Abbvie, and has served on advisory boards for Sanofi. **L. Bouillet** has consulted/served as a speaker for, engaged in research and educational projects with, or accepted travel grants from the following companies: BioCryst, CSL Behring, Takeda, Novartis, GlaxoSmithKline, Blueprint, Intellia, Astra Zeneca, Pharvaris, and Kalvista. **R. Asero** is or recently was a speaker and/or advisor for GlaxoSmithKline, HAL allergy Malesci, Menarini, Lofarma, Thermo Fisher, Novartis, and Sanofi. **M. Gonçalo** has been a speaker and/or advisor for Abbvie, AstraZeneca, Leo Pharma, Lilly, Novartis, Pfizer, Sanofi, and Takeda. **K. Rutkowski** has been a speaker and has served on advisory boards for Novartis. **J. A. Bernstein** has been a Principal Investigator/Consultant for Sanofi‐Regeneron, AstraZeneca, Novartis, Genentech, Amgen, Allakos, Takeda/Shire, CSL Behring, Biocryst, Pharming, Kalvista, Ionis, Pharvaris, Escient, Astria, Celldex, Biomarin, Blueprint Medicine, and Cogent. **Z. Brzoza** has been a speaker for Novartis. **J. I. L. Sousa** has been a speaker for Novartis, Sanofi and Faes Farma. **S. F. Thomsen** has been a speaker or has served on advisory boards for Sanofi, AbbVie, LEO Pharma, Pfizer, Eli Lilly, Novartis, UCB Pharma, Union Therapeutics, Almirall, and Janssen Pharmaceuticals and has received research support from Sanofi, AbbVie, Leo Pharma, Novartis, UCB Pharma, and Janssen Pharmaceuticals, with no relation to the present manuscript. **M. van Doorn** or recently was a speaker and/or advisor for and/or has received research funding from Novartis, AbbVie, Pfizer, LEO Pharma, Sanofi, Lilly, Janssen, UCB, BMS, Celgene, and Third Harmonic outside the submitted work. **J. Peter** has been a speaker or has served on advisory boards for Sanofi, Novartis, Abbivie, Janssen Pharmaceuticals, Takeda, CSL Behring, Pharming, Phavaris, and Astria Therapeutics. **M. Hide** has been a speaker or has served on advisory boards for Kaken Pharmaceutical, Kyowa Kirin, Kyorin, Mitsubishi Tanabe Pharma, Novartis, Sanofi, Taiho Pharmaceutical, Takeda, Teikoku Seiyaku, and Tori. **Y. Ye** has been a speaker for Novartis and has served on advisory boards for Yuhan. **Z. Zhao** is the speaker/advisor for and/or has received research funding from Novartis, Sanofi, Pfizer, Astellas, Galderma, Janssen, GlaxoSmithKline, Bayer, Leo, MEDA Pharma, and ALK Pharma. **I. Nasr** has been a speaker for Novartis, Sanofi, Takeda, and GlaxoSmithKline with no relation to the present manuscript. **H. Rockmann‐Helmbach** has received research funding from Pharming and Novartis Pharma, has received institution fees for advisory board activities from Sanofi, Third Harmonic Bio and Novartis, and has received speaker fees from Novartis Pharma. **R. Ö. Kara** has no conflicts of interest in relation to this manuscript. She has been a speaker for Novartis and Abbvie. **R. Stepanenko** has no conflicts of interest in relation to this manuscript. Outside it, he has conducted studies for/was an advisor for/was a speaker for Alvotech, GlaxoSmithKline, Mitsubishi Tanabe Pharma, Pfizer, Sanofi, and Uriach. **M. Maurer** or recently was a speaker and/or advisor for and/or has received research funding from Allakos, Alvotech, Amgen, Aquestive, Aralez, AstraZeneca, Bayer, Celldex, Celltrion, Evommune, GlaxoSmithKline, Ipsen, Kyowa Kirin, Leo Pharma, Lilly, Menarini, Mitsubishi Tanabe Pharma, Moxie, Noucor, Novartis, Orion Biotechnology, Resonance Medicine, Sanofi/Regeneron, Septerna, Third HarmonicBio, ValenzaBio, Yuhan Corporation, and Zurabio. **S. Neisinger, B. Sousa Pinto, A. Ramanauskaite, C. A. S. Parisi, C. Guillet, L. Han, H Hardin, K. Godse, L. Lapiņa, S. Vandersee, A. Kurchenko, I. Kaidashev, V. Tsaryk, N. Kurjāne** and **J. Astrup Sørensen** declare no conflicts of interest in relation to this work.

## Data Availability

The data that support the findings of this study are available from the corresponding author upon reasonable request.
